# How well do whole exome sequencing results correlate with medical findings? A study of 89 Mayo Clinic Biobank samples

**DOI:** 10.3389/fgene.2015.00244

**Published:** 2015-07-24

**Authors:** Sumit Middha, Noralane M. Lindor, Shannon K. McDonnell, Janet E. Olson, Kiley J. Johnson, Eric D. Wieben, Gianrico Farrugia, James R. Cerhan, Stephen N. Thibodeau

**Affiliations:** ^1^Department of Pathology, Memorial Sloan Kettering Cancer CenterNew York, NY, USA; ^2^Department of Health Sciences Research, Mayo ClinicScottsdale, AZ, USA; ^3^Center for Individualized Medicine, Mayo ClinicRochester, MN, USA; ^4^Department of Health Sciences Research, Mayo ClinicRochester, MN, USA; ^5^Informed DNA, St. PetersburgFL, USA; ^6^Department of Biochemistry and Molecular Biology, Mayo ClinicRochester, MN, USA; ^7^Department of Gastroenterology and Hepatology, Mayo ClinicJacksonville, FL, USA; ^8^Department of Laboratory Medicine and Pathology, Mayo ClinicRochester, MN, USA

**Keywords:** sequencing, exome, EMR, genotype, phenotype, HGMD, OMIM

## Abstract

Whole exome sequencing (WES) is increasingly being used for diagnosis without adequate information on predictive characteristics of reportable variants typically found on any given individual and correlation with clinical phenotype. In this study, we performed WES on 89 deceased individuals (mean age at death 74 years, range 28–93) from the Mayo Clinic Biobank. Significant clinical diagnoses were abstracted from electronic medical record via chart review. Variants [Single Nucleotide Variant (SNV) and insertion/deletion] were filtered based on quality (accuracy >99%, read-depth >20, alternate-allele read-depth >5, minor-allele-frequency <0.1) and available HGMD/OMIM phenotype information. Variants were defined as Tier-1 (nonsense, splice or frame-shifting) and Tier-2 (missense, predicted-damaging) and evaluated in 56 ACMG-reportable genes, 57 cancer-predisposition genes, along with examining overall genotype–phenotype correlations. Following variant filtering, 7046 total variants were identified (~79/person, 644 Tier-1, 6402 Tier-2), 161 among 56 ACMG-reportable genes (~1.8/person, 13 Tier-1, 148 Tier-2), and 115 among 57 cancer-predisposition genes (~1.3/person, 3 Tier-1, 112 Tier-2). The number of variants across 57 cancer-predisposition genes did not differentiate individuals with/without invasive cancer history (*P* > 0.19). Evaluating genotype–phenotype correlations across the exome, 202(3%) of 7046 filtered variants had some evidence for phenotypic correlation in medical records, while 3710(53%) variants had no phenotypic correlation. The phenotype associated with the remaining 44% could not be assessed from a typical medical record review. These data highlight significant continued challenges in the ability to extract medically meaningful predictive results from WES.

## Introduction

The decreasing cost and turn-around time of next generation sequencing (NGS) is accelerating the availability of clinical personal genomes and exomes (Church, 2005; Altshuler et al., 2010). However, data on the predictive clinical utility of whole genome sequencing (WGS) or whole exome sequencing (WES) are minimal, particularly among unselected patients. Also, the capacity for interpretation of the functional consequences of the vast number of variants reported from sequencing data is lagging, but this has not dampened the optimism and expectation that personal WGS/WES will benefit numerous individuals. Many institutions are pursuing this path, anticipating that the evidence supporting this approach will emerge with experience.

A normal individual has been estimated to have approximately 100 loss-of-function mutations and 50–100 mutations in the heterozygous state that can cause a recessive Mendelian disorder as a homozygous genotype (Altshuler et al., 2010; MacArthur et al., 2012). Currently, few large-scale studies have comprehensively evaluated the number of clinically interpretable variants from WES of an individual and how the genetic variants from WES correlate with the medical phenotype of an individual.

The ClinSeq project aims to sequence 1000 subjects in order to determine genotype-phenotype association of variants and modes of returning results to individual subjects (Biesecker et al., 2009; Biesecker, 2012). To date, this group has identified 12 participants (1.2%) with a gene mutation that leads to markedly increased risk of cancer. In a recent study, a Harvard Medical School team utilized published recommendations of the National Heart, Lung, and Blood Institute (NHLBI) group for the return of results (Cassa et al., 2012). They evaluated a representative sample of 160 published disease-associated variants and extrapolated a conservative genome-wide estimate of 3955–12,579 variants per individual to be reported back. The NHLBI recommendations include selecting variants with important health implications where associated risks are established and substantial, genetic finding is actionable, test is analytically valid and proper informed consent has been recorded. In a second study of actionable, pathogenic incidental findings in 1000 WES participants, 114 genes selected by experts for medically actionable conditions were screened in more detail (Dorschner et al., 2013). They reported that 585 of the 1000 participants harbored 239 unique variants identified as disease causing in Human Gene Mutation Database (HGMD). A study from Stanford (Dewey et al., 2014) analyzed 12 WGS samples and highlighted the lack of coverage in some of the 56 American College of Medical Genetics (ACMG) reportable genes and large discordance of INDEL from two sequencing technologies. They curated 90–127 variants per person yielding 2–6 personal disease-risk findings per individual.

To further understand how WES findings might correlate with medical events during a person's life, we conducted WES on 89 individuals from the Mayo Clinic Biobank who were deceased at the time they were selected for sequencing. All 89 had a long history of medical care and extensive medical records at Mayo Clinic, had enrolled in the Mayo Clinic Biobank, and were from the Rochester, Minnesota vicinity. We evaluated the number and characteristics of reportable variants found from WES on this cohort and describe how the variants correlated with medical diagnoses.

## Materials and methods

### Sample selection

The Mayo Clinic Biobank protocol has been approved by the Mayo Clinic Institutional Review Board. All experiments conform to regulatory standards. Informed consent was obtained from all subjects.

The Mayo Clinic Biobank is a research resource that has enrolled over 50,000 Mayo Clinic patient volunteers since 2009 (Olson et al., 2013). Patients at Mayo Clinic who are 18 years or older, English speaking, have mental capacity to consent, and are residents of the USA are eligible for the Mayo Clinic Biobank. Recruitment was conducted via a mailed invitation to people scheduled for an appointment in internal medicine, family medicine, preventive medicine, and the specialty areas of obstetrics/gynecology and executive health. No threshold for health or disease was required to enroll in the Biobank. A blood sample was collected from consented participants providing DNA from white blood cells, serum, plasma and buffy coat.

The first group (group-1) of 39 Biobank participants (Table S1) was selected for WES based on three major criteria: (a) being deceased; (b) long period of electronic medical record (EMR) information (median 15 and mean 13 years); and (c) later age of death. Preference was given to those with a death certificate available at the time of selection to confirm cause of death. Fifty-three deceased subjects were available at the time of the group-1 selection. Nearly all of the confirmed causes of death were due to diseases common in the USA (cancer, heart/lung disease, or trauma) which is consistent with causes of death in the general population of this age group. Of the 39 participants, 23(59%) had a diagnosis of cancer. As further funding became available for the project, the second group (group-2) of 50 Biobank participants (Table S1) was selected. We attempted to diversify the medical diagnoses among this group by preferential selection of individuals without a history of cancer, non-smokers and those with a younger age of death. Since there were not strict inclusion or exclusion criteria, 16(32%) of this group of 50 participants had a diagnosis of cancer. Overall, 39(44%) of the 89 participants had a diagnosis of cancer.

### Patient phenotype

To gain a high-level view of how genotype might correlate with phenotype, a medical geneticist abstracted all significant medical diagnoses from the EMR at Mayo Clinic for each study participant. Of the 89 participants with a mean EMR of 13 years, 55(61%) had more than 15 years of EMR while the remaining 34 had a median EMR of 12 years (inter-quantile range of 8–14 years). Diagnoses were entered into a free-text field. Participants on average had 12 diagnoses (range 2–20). Diagnoses made only as part of the terminal event were not included when they reflected end-of-life situation. Many, but not all participants had seen multiple specialists. Undoubtedly this type of chart audit misses some diagnoses and clinical findings depending on the reasons for each medical visit, but given the routine use of the self-reported past medical illnesses and review of systems forms, the records were fairly comprehensive. The complete chart review of diagnoses for individual participants is not provided in order to avoid recognition in the small community. A representative set of 200 unique diagnoses is listed in Table S2.

### Sample preparation and DNA exome capture

DNA samples from the two groups of Mayo Clinic Biobank participants were sequenced a year apart, based on resources becoming available for WES and analysis. The samples in group-1 (*N* = 39) were captured using Agilent's 50 Mb SureSelect Human All Exon chip, while the group-2 samples (*N* = 50) were captured using Agilent's SureSelect V4 + UTR kit. The enriched DNA samples from the two groups were sequenced as one sample per lane on Illumina Genome Analyzer IIx flow cell and three samples per lane on the Illumina HiSeq 2000, respectively. Sequencing was performed as 101 bp × 2 paired-end reads using the TruSeq SBS sequencing kit version 1 and data collection version 1.1.37.0 followed by base-calling using Illumina's RTA version 1.7.45.0.

### Bioinformatics analysis and annotation

**Figure 1:**
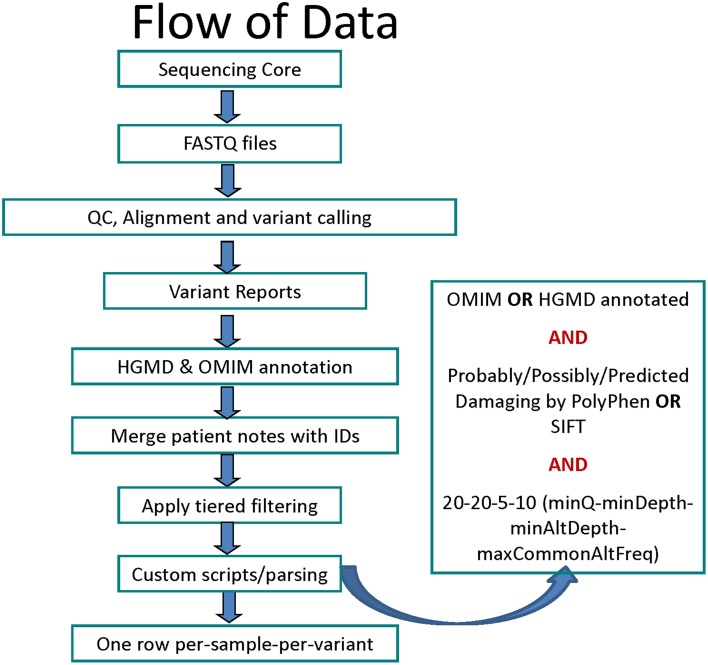
**Flowchart for data analysis stages starting with sequencing to variant calling, filtering and annotation**.

The data was analyzed using an in-house workflow and updated TREAT annotation package (Asmann et al., 2012). Briefly, the sequencing reads were quality checked using FASTQC (Andrews, 2012) and custom tools, aligned using Novoalign (Hercus, 2012), re-aligned and re-calibrated using GATK (McKenna et al., 2010; DePristo et al., 2011), followed by base-quality and variant-quality score recalibration and Single Nucleotide Variant (SNV), Insertion/Deletion (INDEL) calling using GATK (Figure 1). The variants were then annotated using SeattleSeq (Ng et al., 2009, 2012), SIFT (Ng and Henikoff, 2003), PolyPhen (Adzhubei et al., 2010), Variant Effect Predictor and internal annotation databases and reported in VCF and Excel formats. Custom parsing scripts were used to include HGMD v2012.3 (Stenson et al., 2003) and Online Mendelian Inheritance in Man (OMIM) Feb-2013 (Online Mendelian Inheritance in Man, 1998) annotation. The list of data sources used for variant annotation is provided in Table S3.

### Array genotyping

Group-1 samples was genotyped using either the Illumina Infinium HumanOmni2.5-8 plus arrays (*N* = 4) or the Illumina Infinium HumanOmni5-Quad array (*N* = 35); group-2 was genotyped using the Illumina Infinium HumanOmni2.5vv1.1 array (*N* = 50). Concordance rates comparing WES variant calls to array genotypes were calculated for each subject. All WES variant calls with read-depth >10 were included in the concordance analysis.

### Custom variant filtering

Because clinical correlation was an eventual goal, a customized filtering strategy was devised for SNVs. First, only SNVs found in a gene with a listed HGMD or OMIM phenotype were included. SNVs in genes not listed in either database were excluded as those genes have no described clinical consequences (note—this exclusion will make our list of variants smaller than studies that record all variations in DNA in all genes). The exact variant was not required to be reported in OMIM or HGMD. In HGMD, there are a variety of variants and genes that have had some functional work conducted but have not been associated with any disease state and these were removed as uninterpretable. In addition, reported non-disease traits were also removed (Table S4).

SNVs were required to have a minimum (PHRED-scale) mapping quality score of 20 (implying an accuracy >99%), a minimum depth of 20 mapped reads, and a minimum alternate (non-reference allele) read-depth of 5 (Figure 1). The variants with minor allele frequency of ≥ 10% in the 1000 genomes (Abecasis et al., 2012) (phase1 release v3 from Nov 2010), HapMap (Feldman et al., 2013) (v3.3), NHLBI ESP exomes (Fu et al., 2013), or 200 BGI Danish exomes (Li et al., 2010) were excluded. For missense variants, a deleterious *in-silico* prediction was required from either PolyPhen or SIFT. Finally, only SNVs that were defined as follows were included: (1) Tier-1 SNV—gain of stop-codon (nonsense), loss of start-codon or stop-codon, or splice site variants; and (2) Tier-2 SNV—missense variants (with *in-silico* support for pathogenicity).

With respect to INDELs, again only those affecting genes with a listed phenotype in HGMD or OMIM were selected. A minimum alternate (indel supporting) read-depth of 5 reads was required to filter out false positive calls. INDELs were also split by potential impact as: (1) Tier-1 INDEL, frameshift or splice site; and (2) Tier-2 INDEL, codon change or codon deletion/insertion.

### Gene inheritance mode

Prior to evaluating genotype–phenotype correlation, it was necessary to assign each genetic entry to the inheritance pattern generally associated with disorders caused by that gene. For each gene containing a variant included in the Tier-1 or Tier-2 files, a medical geneticist assigned that gene to one of seven groups: (1) autosomal dominant (AD); (2) autosomal dominant or autosomal recessive (AD/AR); (3) autosomal recessive only (AR); (4) X-linked recessive (XLR); (5) X-linked dominant (XLD); (6) Y-linked (YL); and (7) Genome Wide Association Study (GWAS) association only (single nucleotide polymorphism or SNP). Genes whose only known relevance was for containing a SNP of interest per GWAS studies were further subdivided by whether the variant found was an exact match with the associated GWAS SNP or if the variant was in the same gene but was in fact different from the SNP with the known association. Assigning genes to one of these seven groups was a very inexact science as some classical autosomal dominant disorders contain SNV with associations in GWAS for entirely different phenotypes. In general, a good faith effort was made to assign each gene into the most established category for that gene.

### Genotype–phenotype correlation scoring

Once the inheritance pattern and the clinical phenotypes were added to the Tier-1 and Tier-2 variants, a medical geneticist manually scored each genetic variant by comparing the participants' disease phenotypes with all of the phenotypes that had been reported in the gene (not restricted to a specific variant). The phenotype listings were obtained from both HGMD and OMIM and were compared side by side with the patient disease diagnosis list. A “Yes” score meant the participant phenotype overlapped in some way with one of the reported phenotypes for that gene. A “No” meant there was no overlap seen. An “X” indicated inability to assess for genotype–phenotype correlations, for example, a monoallelic change in a recessive gene, gene variant associated with prostate cancer found in a woman; a gene resulting in abnormal sperm shape, which would not have been identified on typical medical visits; or variants that reduced risk for various conditions.

### Coverage analysis of 56 ACMG-reportable and 57 cancer genes

Individual gene level coverage analysis was performed using BEDTools (Quinlan and Hall, 2010) and custom scripts on the aligned BAM files to evaluate efficient reporting of variants from the 56 genes for which clinical reporting has been recommended by the ACMG (ACMG-reportable genes) (Green et al., 2013).

### Cancer-predisposition genes and cancer phenotypes

The subset of genes known to be linked to cancer in the ACMG-reportable list of 56 genes (Green et al., 2013) (*N* = 23), and those now included on some of the cancer-predisposition NGS panels offered clinically (Table S5) were collected (*N* = 34). The resulting 57 genes were used to select all Tier-1 or Tier-2 SNVs and INDELs potentially disrupting the function of these genes. Manual curation was conducted for each variant in this list by a clinical molecular geneticist and variants were scored using the scale: 1 = neutral/non-pathogenic, 2 = likely neutral/non-pathogenic, 3 = variant of uncertain significance; 4 = likely pathogenic, and 5 = pathogenic. The 89 Biobank participants were separated into those with cancer (excluding non-melanoma skin cancers) and those without cancers to compare the genetic results and variant burden.

## Results

### Data metrics

An average of 270 million reads (range of 140–421 million) and 116 million reads (range of 69–147 million) of sequence data were obtained for the group-1 and group-2 samples, respectively (Table 1). The difference in throughput is attributed primarily to differences in the number of samples sequenced per lane; one sample per lane was used for group-1 compared to three samples per lane sequenced for group-2. Approximately 94% (min 87.6, max 96.5) of the targeted region was covered with at least 20 reads (or 20x coverage) for group-2 samples compared to 87.5% (min 83, max 91.8) for group-1 samples. The Agilent SureSelect V4+UTR capture kit used for the group-2 samples had much better capture efficiency with a greater fraction of all sequenced reads mapping to the intended capture region and greater balance and uniformity in the overall coverage of the capture region. Due to the larger capture region in Agilent SureSelect V4+UTR, there were a greater number of variants (SNVs and INDELs) reported for group-2. However, when evaluating the final filtered lists of Tier-1 and Tier-2 variants, there were minimal differences in the number of variants between the two groups (Table 1).

**Table 1:** **Number of reads and variants per sample from the 89 WES individuals**.

	**Total number of variants**	**Group-1^+^ of 39 samples (per-person count)**			**Group-2^+^ of 50 samples (per-person count)**		
		**Mean**	**Max**	**Min**	**Mean**	**Max**	**Min**
Total reads (in millions)		270	421	141	116	147	69
Mapped reads (in millions)		259	405	134	115	146	68
Mapped reads on target (in millions)		134	212	58	92	117	38
% Coverage of targeted region at 5x		94.34	97.3	91.98	98.9	98.93	98.76
% Coverage of targeted region at 10x		91.13	94.7	88.16	98.1	98.78	96.05
% Coverage of targeted region at 20x		87.52	91.8	82.98	94.4	96.52	87.62
Total SNV in the coding regions		42,661	49,065	38,444	64,696	68,875	61,996
Tier-1 = stop gained/lost/start lost/splice site		149	172	134	191	215	169
Tier-1 after filtering^*^	374	4	7	0	4	9	1
Tier-2 = missense		8751	9504	8164	11,039	11,387	10,650
Tier-2 after filtering^*^	5703	65	88	44	63	95	47
Total INDELs in the coding regions		3087	3860	2517	7332	7862	6694
Tier-1 after filtering^*^	270	3	7	0	3	10	0
Tier-2 after filtering^*^	699	6	15	0	9	15	3
Total Tier-1 SNV/INDEL after filtering^*^	644	7	14	0	7	19	1
Total Tier-2 SNV/INDEL after filtering^*^	6402	71	103	44	72	110	50
Total Tier-1 and 2, SNV/INDEL after filtering^*^	7046	78	107	56	79	119	62

*The per-sample data is separated into the two groups in which the actual sequencing was performed. Mean, maximum and minimum metrics are shown for each field*.

+ *Group-1 samples were captured using Agilent 50 Mb capture kit and sequenced one sample per lane while group-2 samples were captured using Agilent V4-UTR capture kit and sequenced three samples per lane*.

* *Filtering metrics are (a) HGMD/OMIM annotation, (b) minimum mapping quality 20*.

*(PHRED-scale), minimum read-depth 20, minimum alternate (non-reference allele) read-depth 5 and the maximum common alternate frequency of 10% (in any of the 1000-genomes, HapMap, BGI or ESP dataset population groups) and (c) in case of Tier-2 SNV, predicted damaging/deleterious by at least one of SIFT or PolyPhen2*.

### Concordance with array genotype calls

Per-sample call rates considering all variant positions in the WES capture region were greater than 94.7% for all samples in group-1 (average = 96.02%, range: 94.70–97.10) and greater than 99.5% for all group-2 samples (average = 99.68%, range 99.50–99.80). The concordance between WES-called SNVs and array-called genotypes was also indicative of high-quality sequencing (average 99.73, range: 99.67–99.76 for all samples in group-1 and average = 99.51, range: 98.62–99.57 for all samples in group-2).

### Genotype–phenotype correlation

The EMR diagnoses obtained for each of the participants and the OMIM/HGMD annotations for each of the genetic variants from the WES data were manually compared. A high-level summary of the proportion of variants which correlated with any known phenotypic finding is shown in Table 2.

**Table 2:** **Summary of the proportion of variants and their correlation with any known phenotypic findings from the chart review**.

**Tier-1 SNV**	**# of variants**	**Match**	**No match**	**Cannot assess**	**Tier-1 INDEL**	**# of variants**	**Match**	**No match**	**Cannot assess**
**VARIANTS IN GENES IN WHICH MUTATIONS CAUSE MENDELIAN DISORDERS**									
AD	60	3	55	2	AD	39	4	34	1
AD/AR	13	1	11	1	AD/AR	17	1	16	0
Digenic	2	0	2	0	Digenic	7	1	6	0
AR	92	2	0	90	AR	84	0	0	84
XLR	11	1	2	8	XLR	0	0	0	0
XLD	2	0	2	0	XLD	0	0	0	0
**VARIANTS IN GENES ASSOCIATED WITH NON-MENDELIAN DISORDERS**									
All SNP^*^	181	11	152	18	Variants^*^	120	4	97	19
Novel SNP in gene	147	10	122	15	Novel variant in gene	114	4	97	13
Exact variant	34	0	17	17	Exact variant	6	0	0	6
Others	13	0	0	13	Others	3	0	0	3
Total	374	18	224	132	Total	270	10	159	101
**Tier-2 SNV**	**# of variants**	**Match**	**No Match**	**Cannot assess**	**Tier-2 INDEL**	**# of variants**	**Match**	**No match**	**Cannot assess**
**VARIANTS IN GENES IN WHICH MUTATIONS CAUSE MENDELIAN DISORDERS**									
AD	914	27	861	26	AD	212	2	179	31
AD/AR	177	15	145	17	AD/AR	6	4	2	0
Digenic	58	4	50	4	Digenic	0	0	0	0
AR	2129	2	0	2127	AR	113	2	0	111
XLR	51	0	12	39	XLR	35	0	11	24
XLD	11	0	8	3	XLD	1	0	1	0
**VARIANTS IN GENES ASSOCIATED WITH NON-MENDELIAN DISORDERS**									
All SNP^*^	2326	107	1897	322	Variants^*^	198	11	161	26
Novel SNP in gene	2170	97	1788	285	Novel variant in gene	198	11	161	26
Exact variant	156	7	116	31	Exact variant	0	0	0	0
Others	37	0	0	37	Others	134	0	0	134
Total	5703	155	2973	2575	Total	699	19	354	326

*Tier-1 variants are most likely to be significant; Tier-2 variants contain many variants of uncertain clinical significance (See text for definitions). Autosomal Dominant (AD) and Recessive (AR), X-linked Dominant (XLD) and recessive (XLR). Two SNP groups were delineated: those previously identified as associated with a phenotype in genome wide association studies (“exact variant”) and novel changes that occurred in a different place in a gene with a known GWAS-associated variant (“novel SNP in gene”) and Single Nucleotide Polymorphism (SNP) identified only in genome wide association studies. SNPs were compiled as separate lists. The category “Others” includes variants that are only seen as Somatic and never Germline or in a gene part of a large deletion interval and thus not known to cause phenotype by itself*.

* *Single nucleotide variants (SNPs) and indel variants are further subdivided into those that occur in a gene for which a phenotype has been described (but not with this variant) and those for which a phenotype has been associated with this exact variant in this gene*.

The majority of medical diagnoses observed for these Mayo Clinic Biobank individuals were common complex genetic disorders, similar to that seen in the general population (atherosclerotic cardiovascular disease, Type 2 Diabetes, obesity, degenerative joint disease, cataracts, osteoporosis, etc.), for which there is little useful genotypic information. Overall, 3% (*N* = 202) of the total 7046 Tier-1 and Tier-2 SNV/INDEL variants had a matching phenotype from clinical chart review while 53% (*N* = 3710) variants did not exhibit a correlating phenotype. The remaining 44% (*N* = 3134) variants were unable to be assessed for genotype-phenotype correlations.

For variants in genes known to have autosomal dominant expression (AD or AD/AR), there were 129 Tier-1 variants (73 SNVs and 56 INDELs) identified. Of these, four Tier-1 SNVs and five Tier-1 INDELs were in genes for which there was a phenotypic match (Table 3). On the other hand, 66 Tier-1 SNVs and 50 Tier-1 INDELs in AD or AD/AR genes did not have an apparent phenotypic match to the individual's medical record (Table S6). Among the 1091 Tier-2 SNVs in AD or AD/AR genes, we observed 42 with phenotypic matches (Table S7) compared with 1006 Tier-2 SNVs with no apparent phenotype match to the individual's medical record.

**Table 3:** **AD genes or AD/AR genes with Tier-1 SNV and with Tier-1 INDEL genotypes for which there was a match (shown in bold) with phenotype in a Biobank participant**.

**Gene Name**	**HGMD and OMIM descriptions**	**Matching phenotype(s)**	**Gender**
*SMAD3*	***Aneurysms***-osteoarthritis syndrome|Aortic aneurysms and dissections with early-onset osteoarthritis|***Osteoarthritis***|Thoracic aortic aneurysms and dissections; Loeys-Dietz syndrome, type 3	Degenerative joint disease, abdominal aortic aneurysm	M
*MSR1*	***Atherosclerosis***, increased risk, association with|Barrett esophagus/esophageal adenocarcinoma|Chronic obstructive pulmonary disease, in smokers, association with|Prostate cancer|Prostate cancer, association with.	Atherosclerosis	F
*TULP3*	***Glaucoma***, primary open angle (due to copy number variant in this gene)	Glaucoma suspect	M
*FLG*	***Eczema*** |Eczema, association with|Eczema, association with and Asthma, association with|Fissured skin on hands of patients without dermatitis|Genetic modifier in pachyonychia congenita|Hand eczema, association|Ichthyosis vulgaris|Peanut allergy, association with|Psoriasis|Psoriasis vulgaris|Psoriasis, increased risk, association…….	Eczema	M
*CFHR5*	***Nephropathy*** |Membranoproliferative glomerulonephritis, association with|Haemolytic uraemic syndrome, susceptibility to|Haemolytic uraemic syndrome, atypical|Glomerulonephritis C3|Factor H-related protein deficiency|Dense deposit disease, reduced risk|Chronic kidney disease after streptococcal infection	Chronic renal failure	F
*CFHR5*	***Nephropathy*** |Membranoproliferative glomerulonephritis, association with|Haemolytic uraemic syndrome, susceptibility to|Haemolytic uraemic syndrome, atypical|Glomerulonephritis C3|Factor H-related protein deficiency|Dense deposit disease, reduced risk|Chronic kidney disease after streptococcal infection	Chronic renal failure	M
*GJB2*	***Non-syndromic hearing loss*** |Knuckle pads, leukonychia, sensorineural deafness|Knuckle pads, hyperkeratosis and deafness|Keratoderma, palmoplantar|Keratitis-ichthyosis-deafness syndrome|Ichthyosiform erythroderma, corneal involvement and deafness|Non-syndromic hearing loss |Oral squamous cell carcinoma|Postnatal permanent childhood hearing impairment|Sensorineural hearing loss|Sensorineural hearing loss, non-syndromic …	Hearing loss	F
*DSC2*	***Arrhythmogenic right ventricular cardiomyopathy*** |Arrhythmogenic right ventricular dysplasia/cardiomyopathy|Cardiomyopathy, dilated	Ventricular tachycardia	F
*GJB4*	Progressive symmetric erythrokeratodermia of Gottron|Erythrokeratodermia variabilis|***Deafness***	Hearing loss	M

*Details on the Tier-1 mutations are found in Supplemental files Tier-1 SNV (Table S10) and Tier-1 Indels (Table S11)*.

We then examined the genotype-phenotype correlation from the perspective of starting with the phenotype that participants presented with and then looking at the presence or absence of variants in presumed genes responsible for those phenotypes. In this analysis, 16 of the 89 participants had none of their phenotypes potentially explained by the filtered WES genotypes (Table S8). For the remaining 73 participants, 146(23%) phenotype matches (average 2 per person, range 1–7 matches) were observed from a total of 636 phenotypes. A maximum of seven phenotypic matches were observed in an individual with eight phenotypes obtained from chart review. The genotypes contributing to phenotype matches were all Tier-2 SNVs in genes with AD or AD/AR inheritance or GWAS SNP candidates (Table S8).

### Clinically significant variants in ACMG-reportable genes

The average base level coverage of the coding region for 56 ACMG genes in the 89 WES samples is shown in Figure S1. Among the list of 56 ACMG-reportable genes, we found an average of 1.8 OMIM/HGMD annotated Tier-1 or Tier-2 filtered variants (range 0–6, median 2) per individual. Fifteen individuals had no variants. The 161 variants (13 Tier-1 and 148 Tier-2) found in 74 of Biobank samples involved 27 of the 56 ACMG genes. The 8 Tier-1 SNVs are from five genes (*APOB, BRCA2, LDLR, MYBPC3, SMAD3*) and consist of seven stop-gain and one splice variant from seven samples. The 5 Tier-1 INDELs (in five samples) are all frame-shift from four genes (*BRCA2, DSC2, PCSK9, DSP*). The *BRCA2* variants included a nonsense SNV (p.K3326^*^, noted to be a low-penetrance disease-associated polymorphism), and a frame-shift insertion at c.100096 (which is also considered non-pathogenic, as truncating mutations proximal to this are benign or at least hypo-morphic). All these variants were reported as heterozygous. No confirmatory testing was conducted on any variant.

### Evaluation of variants in cancer-predisposition genes

**Figure 2:**
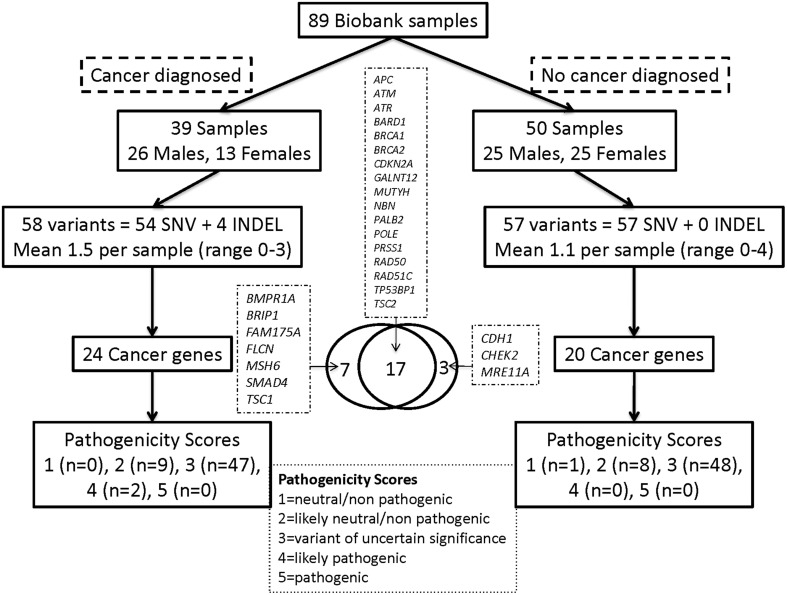
**Distribution of Tier-1/Tier-2 variants by cancer history and gender for the cancer predisposition genes**. Overall, 39 (44%) of the 89 participants had a diagnosis of cancer: 23 (59%) of these from the 39 group-1 participants and 16 (32%) of the 50 group-2 participants.

We also examined the frequency of variants among 57 cancer-predisposition genes (23 from the ACMG list), as defined in the Materials and Methods. The average base level coverage of the coding region for 56 ACMG genes in the 89 WES samples is shown in Figure S2. A total of 115 genetic Tier-1 or Tier-2 variants were found using our custom variant filtering. The distribution of these variants by cancer history and gender is shown in Figure 2 and Table S9. Overall, 39(44%) of the 89 participants had a diagnosis of cancer: 23(59%) of these were from the 39 group 1 participants and 16(32%) from the 50 group two participants. There were an average of 1.3 (range 0–3) variants per subject with cancer and about 1.1 (range 0–4) variants in subjects without cancer. After manual curation of pathogenicity scores, there were two variants in the subjects with cancer with a score of 4 (likely pathogenic) compared to none in the subjects without cancer (Fisher's exact test *p* = 0.19). These two variants, both heterozygous, are a missense SNV in *BRIP1* and a frame-shift mutation in *ATR*.

Tables S10–S13 show genetic details of the variant calling of all types and Table S14 shows details of the Tier-1 gene variants for which no phenotypic matches were evidence by medical record review.

## Discussion

This study evaluated potentially significant coding region DNA variants in genes of reported clinical significance. Our goal was to examine the genotype–phenotype correlation from WES studies in a series of individuals representing a broad range of phenotypes. WES and systematic interpretation was carried out on 89 individuals who had lived out their entire lifespan and whose medical records were available for correlation with WES genotypes. We selected a set of samples from the Mayo Clinic Biobank that itself moderately represents the general population (Olson et al., 2013). Overall, there were 51 males and 38 females with an average age at death of 74.5 years (range 28–93, median 78 years). In comparison, the average age of death in the US is 79, 80.9 years in the state of Minnesota and 82.4 years in Olmsted County (Olmsted County Public Health Service Records, 2014) where Rochester, MN is located. As expected, a majority of their medical diagnoses were those of the general population (atherosclerotic cardiovascular disease, Type 2 Diabetes, obesity, degenerative joint disease, cataracts, osteoporosis, etc.) and were not accounted for by highly penetrant Mendelian gene variants. The contribution of WES in providing information that allows people to be proactive for these multifactorial disorders is understood to be minimal. Of more interest to this study, however, was the degree to which mutations in genes of more Mendelian/single gene disorders did or did not correlate with medical events in these individuals' lifetimes.

Overall, a total of 374 Tier-1 SNVs (stop codon-gain/loss, start codon-loss or splice altering) were identified following our filtering strategy. Among these, only four SNVs (3.1%) and five INDELs (3.8%) out of 129 variants in AD or AD/AR genes known to have autosomal dominant expression were found to have a phenotypic match (Table 3). The first variant for which a phenotypic match was identified is a novel splice variant in *SMAD3* gene. While the affected individual did not have a Loeys–Dietz phenotype, he did have a small abdominal aortic aneurysm and degenerative joint disease in his 60s. In addition, he had idiopathic pulmonary fibrosis, which has not been associated with *SMAD3* in humans. However, animal studies have suggested a role for *SMAD*3 in fibrosing disorders in mice (Gauldie et al., 2006; Warburton et al., 2013). Had this variant been discovered during the individual's lifetime, it would have been concerning, but optimal clinical management is unknown. Of the other three SNVs with overlap between gene and patient phenotype, the Tier-1 *FLG* mutation p.R501^*^ (Table 3, Table S10) likely contributes significantly to the person's eczema. Regarding genes associated with atherosclerosis and glaucoma, it is unlikely that the identified gene variants were major contributors to these common and complex phenotypes and prior knowledge of these variants would not likely have led to specific medical interventions beyond typical medical care.

Of the 1091 Tier-2 SNV (missense predicted-damaging) identified in AD or AD/AR genes, 42(4%) demonstrated a clinical correlation (Table S7). However, most of these are multifactorial disorders and the match called out is unlikely to be a major cause. Two exceptions to this may be the *RUNX1* mutation in an individual with myelodysplastic disorder and the *MC1R* mutation in an individual with two melanomas. These variants were found in 0 and 2 other Biobank individuals, respectively with a frequency of 0% and 1.7% within the 1000 Genomes dataset, respectively, and so are not common and may be important in these individuals. All of the Tier-2 SNV variants in recessive genes were found only as a single copy, and thus carriers only. There were no homozygotes or compound heterozygotes.

On the other hand, the list of Tier-1 SNVs in AD or AD/AR genes for which no phenotypic match was apparent (66 of 73, 90%) was much longer (Table S6). Even if some of these genes are not well-established as disease causative or reported to have lower penetrance than typical Mendelian, it is still notable that the list of variants in genes with no evident phenotype is multiple times larger than the list of genes with phenotypic matches. A clinician undertaking testing of an individual would not know which of the Tier-1 SNV might actually be relevant to this person and which would not.

The majority of Tier-2 variants were classified as having uncertain significance using draft guidelines presented at the 2013 National Society of Genetic Counselors (NSGC) meeting (final guidelines and manuscript were not available when this study done). Although these guidelines, presented by a member of the ACMG task force working on variant classification, deemed *in-silico* analysis alone as insufficient to distinguish pathogenic from non-pathogenic variants, we used *in-silico* calls to include “damaging” variants in this correlation study. We observed a stark contrast between the large number of variants discovered and the low number of times for which a phenotypic match, even very leniently defined, could be found (Table 2). In this dataset, there were thousands of variants that have been reported in OMIM/HGMD genes that *in-silico* analysis defined as likely damaging, but the evidence for that effect in the lives of these individuals was absent in the vast majority of instances.

We observed a similar trend of few matches when viewing the genotype–phenotype correlation from the perspective of assessing the phenotypes available from chart review with the genotype from WES analysis. None of the phenotypes from 16 of the 89 individuals had a match. For the remaining individuals, approximately two from an average of nine phenotypes per individual matched the WES analyzed genotypes (Table S8). Moreover, a majority of these matches were GWAS SNPs that would be expected to make minor contributions to the phenotypes.

We also evaluated the 56 genes for which clinical reporting was recommended by the ACMG (Green et al., 2013). We found a median of 2 (range 0–6) OMIM/HGMD annotated Tier-1 or Tier-2 filtered variants per individual among the list of 56 ACMG-reportable genes. Of the 89 participants, 15 had no variants in ACMG-reportable genes. These numbers are comparable to median of 3 (range 1–7) potentially pathogenic variants found in 12 WGS samples reported by Dewey et al. (2014).

Because the presence or absence of cancer diagnosis is more straightforward to categorize from chart review than other medical disorders (e.g., limited ability to determine if diagnoses like cardiomyopathy or renal failure are primary or secondary on most chart reviews), a deeper evaluation of Mendelian cancer-predisposition genes was conducted. There were no significant differences in the number of filtered variants per individual identified in cancer-predisposition genes in individuals with or without cancer (Table S9). After manual assignment of pathogenicity scores, there were two variants in subjects with cancer with a score of 4 (likely pathogenic) compared to none in the individuals without cancer. Presently, our ability to determine which DNA variants are pathogenic and which are benign is a major limiting factor in tapping into the clinical utility of WES. This sub-analysis of cancer genes does suggest that a subset of the genetic variants might be contributing to disease, but that most missense variants, which were present in similar numbers in those with and without cancer diagnoses, are not creating apparent risk.

Our WES dataset of 89 samples generated on average 79 (range 56–119) filtered variants (SNV and INDEL) per individual (Table 1). Correspondingly, a median of 108 (range 90–127) variants (including SNV, INDEL and structural changes) per sample were identified from a WGS study of 12 individuals (Dewey et al., 2014). Although not largely different, varying sequencing coverage and stringency of filtering methods used are likely to be the reason for differences between WES and WGS results. For instance, an important step to identify local artifacts from bioinformatics analysis is to filter frequently reported variants. This step was performed for our data removing more than 30% of the called variants by filtering variants seen in 10% or more of the 89 WES samples. The 12 sample WGS dataset (Dewey et al., 2014) was too small to take advantage of the filtering.

Notable challenges of this analytic approach include personnel time needed for manual literature review, the subjective nature of bioinformatics filtering thresholds, and uncertainty about variant pathogenicity. Though not timed, we would agree with recent reports that expert review of each variant to score for pathogenicity could take around an hour per variant (Dewey et al., 2014). Despite stringent bioinformatics filtering there are a large number of variants, especially missense, requiring classification. Working groups of experts in genomic research, analysis and clinical diagnostic sequencing are collaboratively looking for recommendations and guidelines for investigating genetic variants' causality in human disease (MacArthur et al., 2014) and databases of curated variants are needed even more urgently than ever as WES/WGS launches.

Our study has a number of important limitations, including the following. One of the technical aspects of this study, and in WES studies in general, is the missing coverage of important genes. An average of 9(17%), with a range 4–17(7–30%) out of the 56 ACMG-reportable genes had sub-optimal coverage per individual for efficient variant calling in our WES data even if the coverage was dropped from 20x (lower cut-off used for the study) to 10x. This study was also limited to SNV and small INDEL identified from WES data. Compared to WGS, WES is not optimal for detecting Copy Number Variation (CNV) and large structural variants. Most of the available tools suffer from limited power to detect CNVs (Tan et al., 2014). Our project involved a single medical geneticist expert evaluating the gene inheritance and pathogenicity classification as opposed to a group of experts engaged in other studies (Dorschner et al., 2013; Dewey et al., 2014). The EMR at Mayo Clinic may have omitted some important diagnoses as patients may have received care elsewhere and not recorded significant findings on their intake forms. The bioinformatics tools used in this study are not clinically validated and arbitrary quality and read-depth thresholds were used for data filtering. The data we analyzed are from self-reported Caucasian individuals only. Our filtering had a heavy reliance on HGMD and OMIM for gene filtering and initial pathogenic mutation identification. Multiple genes whose functions remain unknown were excluded from this study. A large number of the variants assigned *in-silico* as pathogenic may be neutral. To develop a disorder, multiple genes may need to be involved—single gene disorders may be rather rare in reality.

In spite of these limitations, however, this study provides new insights and begins to quantitate the limited correlation between DNA variants and clinical manifestations on an individual basis, and as such, provides a cautionary note regarding the current predictive value of most DNA variants in the setting of a non-disease selected population. The many technical challenges likely affecting the results are unlikely to account for the gap between variants found and absent medical diagnoses. Resolving and understanding these issues will require sustained and large-scale collaborative research.

### Conflict of interest statement

The authors declare that the research was conducted in the absence of any commercial or financial relationships that could be construed as a potential conflict of interest.

## Supplementary material

Figure S1 **Average base level coverage of the coding region for 56 ACMG genes in the 89 WES samples**. Boxplots show the per-base coverage for each gene. The box itself shows the limits of the middle half of the observed data with the top and bottom of the box representing the 75th and 25th percentile and the line inside the box represents the median value. Whiskers are drawn to the nearest values inside of 1.5^*^IQR where IQR = Interquartile Range = 75–25% and values outside of 1.5^*^IQR are displayed as circles.

Figure S2 **Average base level coverage of the coding region for 57 cancer genes in the 89 WES samples**. See Figure S1 for description of box plot.

Table S1 **Age and gender information of the 89 WES Biobank samples**. The age information is shown by gender and also by group, as the 89 samples were sequenced in two groups or batches based on availability of resources and funding.

Table S2 **Representative set of 200 unique diagnoses abstracted from chart review of the 89 participants**.

Table S3 **Sources used for variant annotation**. The collection of various resources used and split by the type of information queried from the annotation sources.

Table S4 **Representative set of 100 non-disease entries in HGMD/OMIM excluded from further evaluation**.

Table S5 **List of 57 cancer-related genes evaluated for the 89 WES samples**. The three columns denote binary presence or absence of these cancer pre-disposition genes in the various clinical NGS gene panels, the list of 56 ACMG-reportable genes and other genes selected based on our experience.

Table S6 **AD genes or AD/AR genes that are either dominant or recessive, with Tier-1 SNV variants for which there was No match with phenotype (***n*** = 55 examples)**.

Table S7 **Number of autosomal dominant genes or dominant/recessive genes with Tier-2 single nucleotide variants for which there was a match (shown in bold) with phenotype in a Biobank participant**. A total of 1091 variants of this type were noted and this was the subset with any phenotypic overlap or match. The other 1006 are not shown.

Table S8 **Phenotypes of 89 individuals from chart review and the matching genotypes from WES data along with variant type, affected gene and gene inheritance information**. T1S, Tier-1 SNV; T2S, Tier-2 SNV; T1I, Tier-1 INDEL; T2I, Tier-2 INDEL; CAD, coronary artery disease; DM2, diabetes mellitus type 2; CHF, congestive heart failure; CM, cardiomyopathy; SCC/BCC, squamous/basal cell carcinoma; AAA, abdominal aortic aneurysm; HCM, hypertrophic cardiomyopathy; BP, blood pressure.

Table S9 **Distribution of 89 WES samples by cancer diagnosis and gender**. Also included are metrics on Tier-1/Tier-2 SNV and INDEL along with the list of cancer predisposition genes found in the groups.

Table S10 **Genetic details for all Tier-1 SNVs**.

Table S11 **Genetic details for all Tier-1 indels**.

Table S12 **Genetic details for all Tier-2 SNV**.

Table S13 **Genetic details for all Tier-2 indels**.

Table S14 **Tier-1 SNVs and indels for which no phenotypic match was evident**.
