# Supplementary figures and images for: How well do whole exome sequencing results correlate with medical findings? A study of 89 Mayo Clinic Biobank samples
Source: Front Genet. 2015 Jul 24;6:244. doi: 10.3389/fgene.2015.00244 (PMC4513238; doi:10.3389/fgene.2015.00244)

Overall coverage on coding regions for ACMG-reportable gene in the 89 WES samples

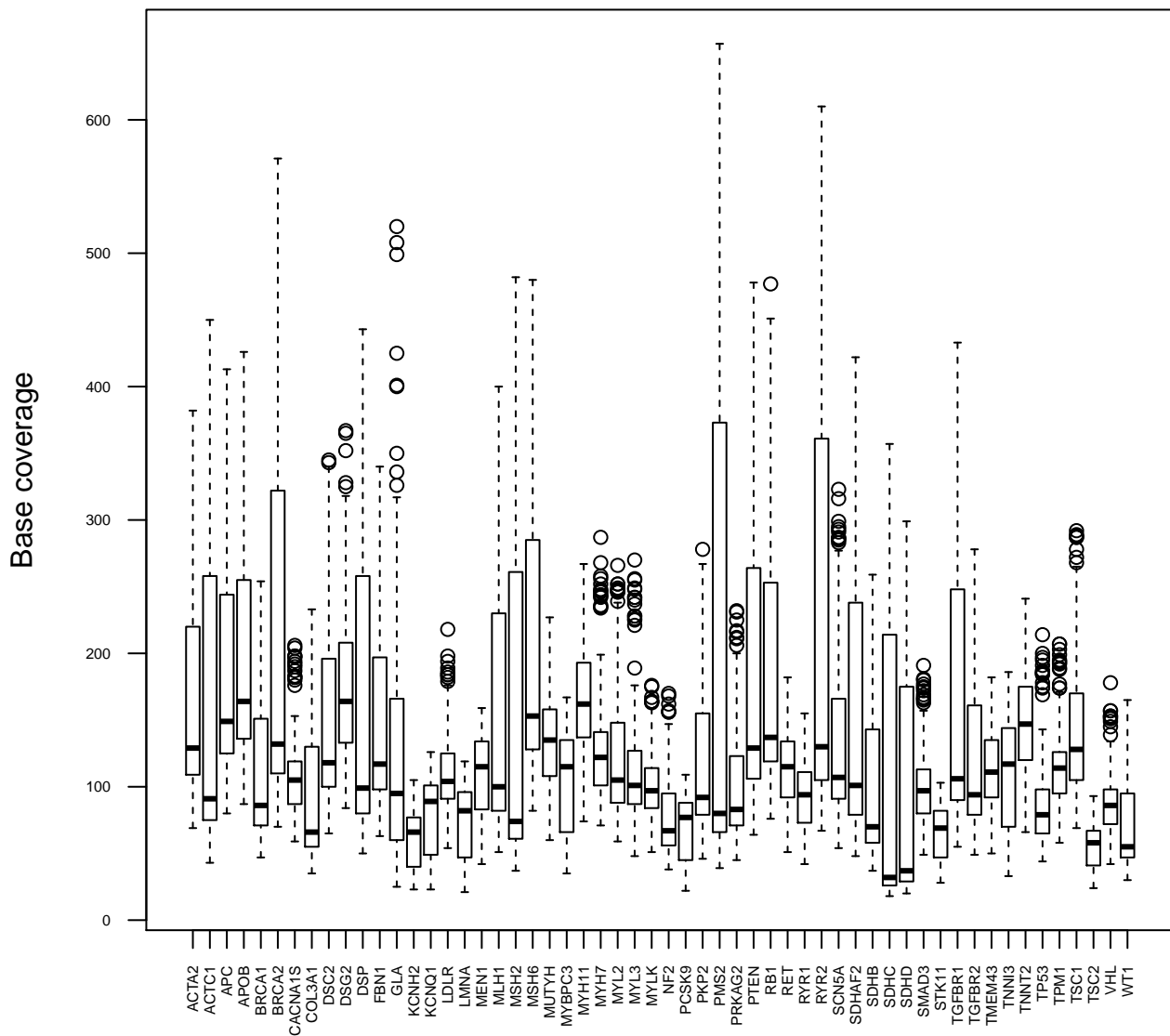

Supplement: Figure S1 — Average base level coverage of the coding region for 56 ACMG genes in the 89 WES samples. Boxplots show the per-base coverage for each gene. The box itself shows the limits of the middle half of the observed data with the top and bottom of the box representing the 75th and 25th percentile and the line inside the box represents the median value. Whiskers are drawn to the nearest values inside of 1.5*IQR where IQR = Interquartile Range = 75–25% and values outside of 1.5*IQR are displayed as circles. [file Image1.PDF]

Overall coverage on coding regions of 57 genes in the 89 WES samples

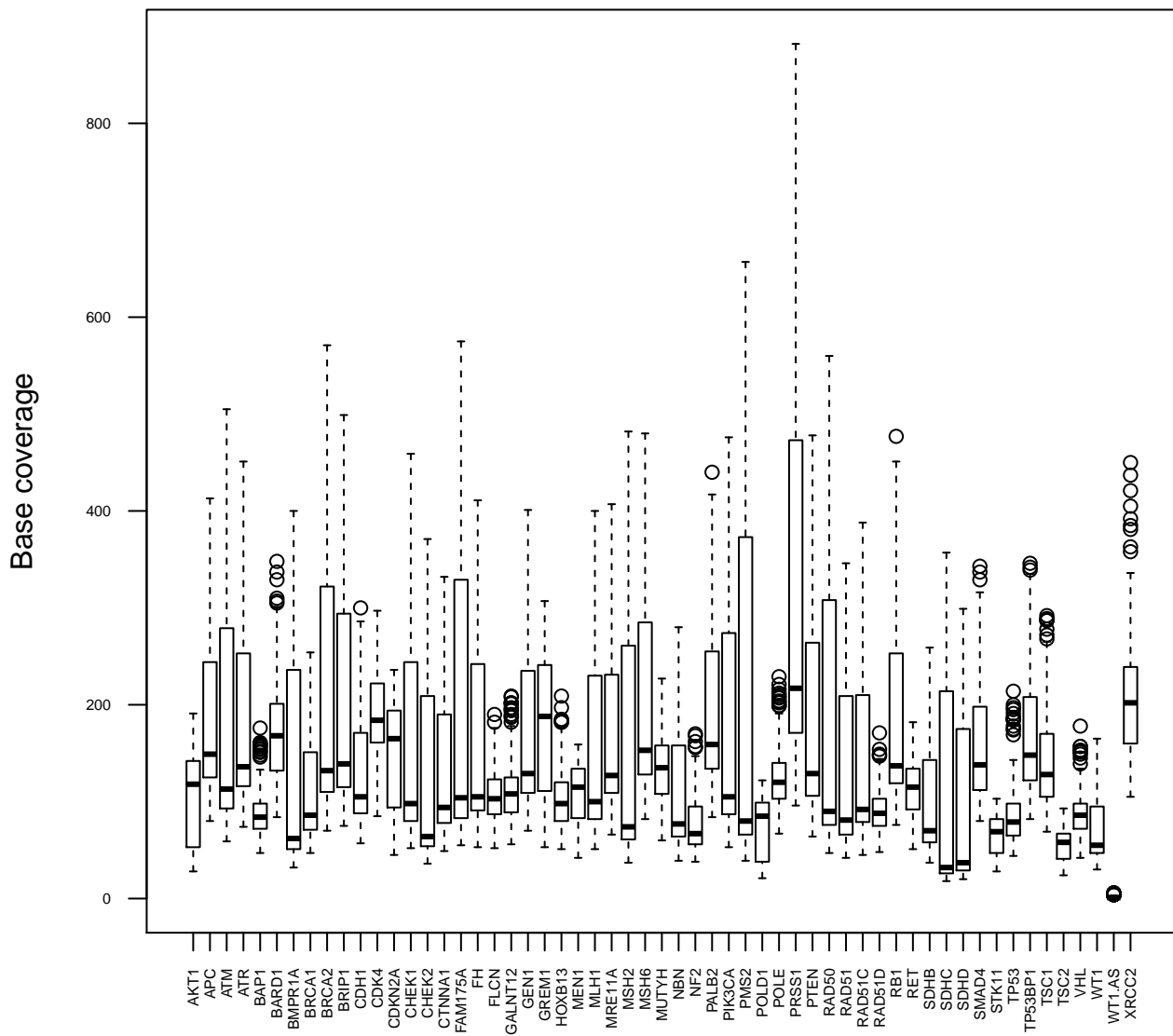

Supplement: Figure S2 — Average base level coverage of the coding region for 57 cancer genes in the 89 WES samples. See Figure S1 for description of box plot. [file Image2.PDF]
