# Supplementary material for: How well do whole exome sequencing results correlate with medical findings? A study of 89 Mayo Clinic Biobank samples
Source: Front Genet. 2015 Jul 24;6:244. doi: 10.3389/fgene.2015.00244 (PMC4513238; doi:10.3389/fgene.2015.00244)
Supplement: Table S1 — Age and gender information of the 89 WES Biobank samples. The age information is shown by gender and also by group, as the 89 samples were sequenced in two groups or batches based on availability of resources and funding. [file Table1.DOCX]

**S1 Table**: Age and gender information of the 89 WES Biobank samples. The age information is shown by gender and also by group, as the 89 samples were sequenced in two groups or batches based on availability of resources and funding.

| **88 Biobank WES samples** | | | |
| --- | --- | --- | --- |
| 74 years at death (range 28-93) | | | |
| **51 Males** | | **38 Females** | |
| 75 years at death | | 74 years at death | |
| 32-91 years at death | | 28-93 years at death | |
| **Group-1** | | **Group-2** | |
| **39 samples** | | **50 samples** | |
| ~77 years at death | | ~72 years at death | |
| 53-93 years at death | | 28-93 years at death | |
| 24 Males | 15 Females | 27 Males | 23 Females |
