# Supplementary material for: How well do whole exome sequencing results correlate with medical findings? A study of 89 Mayo Clinic Biobank samples
Source: Front Genet. 2015 Jul 24;6:244. doi: 10.3389/fgene.2015.00244 (PMC4513238; doi:10.3389/fgene.2015.00244)
Supplement: Table S3 — Sources used for variant annotation. The collection of various resources used and split by the type of information queried from the annotation sources. [file Table3.DOCX]

**S3 Table**: Sources used for Variant Annotation. The collection of various resources used and split by the type of information queried from the annotation sources

| **Data Sources used for variant annotation** | | |
| --- | --- | --- |
| **Allele Frequency estimation** | | |
| NCBI database of Single Nucleotide Polymorphisms database, version 135 | | |
| Hapmap 2 and 3 Utah residents with Northern & Western European ancestry from CEPH collection | | |
| Hapmap 2 and 3 Youruba in Ibadan, Nigeria | | |
| Hapmap 3 and 3 Han Chinese in Beijing, China | | |
| 1000 genomes project phase 1, European ancestry | | |
| 1000 genomes project phase 1, West African ancestry | | |
| 1001 genomes project phase 1, East Asian ancestry | | |
| National Heart Lung Blood Institute Exome Sequencing Project 5400 European Americans | | |
| National Heart Lung Blood Institute Exome Sequencing Project 5400 African Americans | | |
| 200 Danish Exomes sequenced at BGI | | |
| **Gene Annotation** | | |
| ENSEMBL Gene ID | | |
| NCBI RefSeq | | |
| ENTREZ Gene ID | | |
| UCSC known gene | | |
| **Functional Effect Prediction** | | |
| Sorting Intolerant From Tolerant (SIFT) | | |
| PolyPhen2 | | |
| SNP Effect Predictor | | |
| **Phenotype Association** | | |
| Human Gene Mutation Database (HGMD) | | |
| Online Mendelian Inheritance in Man (OMIM) | | |
| NHGRI Genome Wide Association Study catalog | | |
| Catalog Of Somatic Mutations In Cancer (COSMIC) | | |
| CLINVAR | | |
| Leiden Open Variation Database (LOVD) | | |
| Exome Variant Server | | |
| ALAMUT | | |
| Breast Cancer Information Core | | |
