# Supplementary material for: How well do whole exome sequencing results correlate with medical findings? A study of 89 Mayo Clinic Biobank samples
Source: Front Genet. 2015 Jul 24;6:244. doi: 10.3389/fgene.2015.00244 (PMC4513238; doi:10.3389/fgene.2015.00244)
Supplement: Table S5 — List of 57 cancer-related genes evaluated for the 89 WES samples. The three columns denote binary presence or absence of these cancer pre-disposition genes in the various clinical NGS gene panels, the list of 56 ACMG-reportable genes and other genes selected based on our experience. [file Table5.DOCX]

**S5 Table**. List of 58 cancer related genes evaluated for the 89 WES samples. The three columns denote binary presence or absence of these cancer pre-disposition genes in the various clinical NGS gene panels, the list of 56 ACMG-reportable genes and other genes selected based on our experience.

| **GeneID** | **University of Washington BROCA panel** | **ACMG** | **Other** |
| --- | --- | --- | --- |
| *AKT1* | 1 | 0 | 0 |
| *APC* | 1 | 1 | 0 |
| *ATM* | 1 | 0 | 0 |
| *ATR* | 1 | 0 | 0 |
| *BAP1* | 1 | 0 | 0 |
| *BARD1* | 1 | 0 | 0 |
| *BMPR1A* | 1 | 0 | 0 |
| *BRCA1* | 1 | 1 | 0 |
| *BRCA2* | 1 | 1 | 0 |
| *BRIP1* | 1 | 0 | 0 |
| *CDH1* | 1 | 0 | 0 |
| *CDK4* | 1 | 0 | 0 |
| *CDKN2A* | 1 | 0 | 0 |
| *CHEK1* | 1 | 0 | 0 |
| *CHEK2* | 1 | 0 | 0 |
| *CTNNA1* | 1 | 0 | 0 |
| *FAM175A* | 1 | 0 | 0 |
| *GALNT12* | 1 | 0 | 0 |
| *GEN1* | 1 | 0 | 0 |
| *GREM1* | 1 | 0 | 0 |
| *HOXB13* | 1 | 0 | 0 |
| *MLH1* | 1 | 1 | 0 |
| *MRE11A* | 1 | 0 | 0 |
| *MSH2* | 1 | 1 | 0 |
| *MSH6* | 1 | 1 | 0 |
| *MUTYH* | 1 | 1 | 0 |
| *NBN* | 1 | 0 | 0 |
| *PALB2* | 1 | 0 | 0 |
| *PIK3CA* | 1 | 0 | 0 |
| *PMS2* | 1 | 1 | 0 |
| *POLD1* | 1 | 0 | 0 |
| *POLE* | 1 | 0 | 0 |
| *PRSS1* | 1 | 0 | 0 |
| *PTEN* | 1 | 1 | 0 |
| *RAD50* | 1 | 0 | 0 |
| *RAD51* | 1 | 0 | 0 |
| *RAD51C* | 1 | 0 | 0 |
| *RAD51D* | 1 | 0 | 0 |
| *RET* | 1 | 1 | 0 |
| *SDHB* | 1 | 1 | 0 |
| *SDHC* | 1 | 1 | 0 |
| *SDHD* | 1 | 1 | 0 |
| *SMAD4* | 1 | 0 | 0 |
| *STK11* | 1 | 1 | 0 |
| *TP53* | 1 | 1 | 0 |
| *TP53BP1* | 1 | 0 | 0 |
| *VHL* | 1 | 1 | 0 |
| *XRCC2* | 1 | 0 | 0 |
| *MEN1* | 0 | 1 | 0 |
| *RB1* | 0 | 1 | 0 |
| *SDHARF2* | 0 | 1 | 0 |
| *TSC1* | 0 | 1 | 0 |
| *TSC2* | 0 | 1 | 0 |
| *WT1* | 0 | 1 | 0 |
| *NF2* | 0 | 1 | 0 |
| *FLCN* | 0 | 0 | 1 |
| *FH* | 0 | 0 | 1 |
