# Supplementary material for: How well do whole exome sequencing results correlate with medical findings? A study of 89 Mayo Clinic Biobank samples
Source: Front Genet. 2015 Jul 24;6:244. doi: 10.3389/fgene.2015.00244 (PMC4513238; doi:10.3389/fgene.2015.00244)
Supplement: Table S6 — AD genes or AD/AR genes that are either dominant or recessive, with Tier-1 SNV variants for which there was No match with phenotype (n = 55 examples). [file Table6.DOCX]

**S6 Table:** AD genes or AD/AR genes that are either dominant or recessive, with Tier-1 SNV variants for which there was No match with phenotype (n=55 examples)

| **Gene** | **HGMD and/or OMIM descriptions (some truncated)** | # times variants in this gene seen in Biobank 89 |
| --- | --- | --- |
| *AADAC* | Tourette syndrome \|Reduced enzyme activity | 1 |
| *ALK* | Neuroblastoma | 1 |
| *ANO7* | Glaucoma, primary congenital | 1 |
| *ASXL1* | Bohring-Opitz syndrome\|Systemic mastocytosis with associated non-mast cell lineage disease | 2 |
| *BCMO1* | Hypercarotenemia and hypovitaminosis A\|Altered beta-carotene metabolism, association with | 1 |
| *CARD14* | Psoriasis, association with\|Psoriasis\|Pityriasis rubra pilaris | 1 |
| *CATSPER2* | Asthenoteratozoospermia & deafness, non-syndromic | 1 |
| *COL8A2* | Glaucoma, primary open angle\|Fuchs corneal dystrophy | 1 |
| *COMP* | Pseudoachondroplasia\|Multiple epiphyseal dysplasia\|Early-onset osteoarthritis | 1 |
| *CRYBA4* | Cataract and microcornea\|Cataract, lamellar\|Microphthalmia | 1 |
| *DPP6* | Autism spectrum disorder \|Ventricular fibrillation, idiopathic | 1 |
| *EFHC1* | Myoclonic epilepsy, juvenile\|Intractable epilepsy of infancy\|Idiopathic epilepsy, generalised | 1 |
| *FAM83H* | Amelogenesis imperfecta, hypocalcified\|Amelogenesis imperfecta, hypoplastic local | 1 |
| *FREM1* | Bifid nose, renal agenesis & anorectal malformations syndrome\|Craniosynostosis, isolated metopic\|Manitoba-oculo-tricho-anal syndrome | 1 |
| *GON4L* | Intellectual disability | 1 |
| *HBM* | Thalassaemia alpha | 1 |
| *KRT83* | Monilethrix | 12 |
| *MSR1* | Atherosclerosis, increased risk, association with\|Barrett oesophagus/oesophageal adenocarcinoma\|Chronic obstructive pulmonary disease, in smokers, association with\|Prostate cancer\|Prostate cancer, association with | 4 |
| *MYBPC3* | Hypertrophic cardiomyopathy with inclusion body myositis\|Increased left ventricular wall thickness\|Left ventricle dysfunction in CAD, association with\|Skeletal myopathy, association with\|Sudden infant death syndrome \|Dilated cardiomyopathy\|Cardiomyopathy, left-ventricular noncompaction\|Cardiomyopathy, left ventricular noncompaction\|Cardiomyopathy, hypertrophic/dilated\|Cardiomyopathy, hypertrophic\|Cardiomyopathy, dilated\|Cardiomyopathy, association with\|Cadiomyopathy, dilated | 4 |
| *MYO1A* | Sensorineural deafness, nonsyndromic | 1 |
| *NBAS* | Short stature, optic atrophy & Pelger-Huet | 1 |
| *NOL3* | Cortical myoclonus | 1 |
| *OBSCN* | Cardiomyopathy, hypertrophic\|Glioblastoma\|Potential protein deficiency | 1 |
| *PITPNM3* | Cone dystrophy, autosomal dominant\|Cone dystrophy | 1 |
| *PLCB4* | Auriculocondylar syndrome | 1 |
| *POLR1C* | Treacher-Collins syndrome | 1 |
| *PRPH* | Amyotrophic lateral sclerosis\|High myopia | 1 |
| *RAD21* | Cornelia de Lange-like syndrome | 1 |
| *RASA1* | 5q14.3 neurocutaneous syndrome\|Arteriovenous fistula\|Arteriovenous malformation \|Capillary malformation-arteriovenous malformation\|Capillary malformations\|Sturge-Weber syndrome | 1 |
| *RNASEL* | Ribonuclease L deficiency, association with\|Ribonuclease L deficiency\|Prostate, cancer, protection against, association with\|Prostate cancer, association with \|Prostate cancer | 1 |
| *RP1L1* | Macular dystrophy, occult\|Potential protein deficiency | 1 |
| *SLC6A2* | Reduced gene expression\|Orthostatic intolerance and tachycardia\|Major depression\|Decreased transport activity\|Attention-deficit hyperactivity disorder, association with | 1 |
| *TBC1D4* | Insulin resistance | 1 |
| *TRPA1* | Episodic pain syndrome\|Paradoxical heat sensation, association with | 1 |
| *TRPM2* | Amyotrophic lateral sclerosis and parkinson disease | 1 |
| *TTF2* | Autism | 1 |
| *TTN* | Tibial muscular dystrophy\|Potential protein deficiency\|Myopathy with early respiratory failure\|Myopathy with cellular aggregates\|Myopathy\|Muscular dystrophy \|Cardiomyopathy, hypertrophic\|Cardiomyopathy, dilated\|Arrhythmogenic right ventricular cardiomyopathy | 1 |
| *AADAC* | Tourette syndrome \|Reduced enzyme activity | 1 |
| *ALK* | Neuroblastoma | 1 |
| *FLG* | Eczema \|Eczema, association with\|Eczema, association with and Asthma, association with\|Fissured skin on hands of patients without dermatitis\|Genetic modifier in pachyonychia congenita\|Hand eczema, association\|Ichthyosis vulgaris\|Peanut allergy, association with\|Psoriasis\|Psoriasis vulgaris\|Psoriasis, increased risk, association … | 4 |
| *SH3TC2* | Charcot-Marie-Tooth disease 1\|Charcot-Marie-Tooth disease 4C\|Hereditary motor & sensory neuropathy | 1 |
| *VWF* | Von Willebrand disease 2n/1\|Von Willebrand disease 2n\|Von Willebrand disease 2m \|Von Willebrand disease 2c\|Von Willebrand disease 2b-like\|Von Willebrand disease 2b\|Von Willebrand disease 2u\|Von Willebrand disease 3 \|Von Willebrand disease, association with\|Von Willebrand disease, quantitative type, association with\|Von Willebrand, … | 1 |
| *SEMA3E* | CHARGE syndrome | 1 |
| *APOB* | Hypobetalipoproteinaemia\|Hypobetalipoproteinemia-induced nonalcoholic steatohepatitis\|Hypocholesterolaemia \|Hypocholesterolaemia, association with\|Increased apoB and cholesterol levels, association with\|Increased cholesterol levels\|Ischaemic stroke, association with \|Oligoasthenoteratozoospermia, association with\|Hypertriglyceridaemia \|Hypercholesterolaemia \|Altered APOB levels \|Aortic stenosis, association with\|Apolipoprotein B deficiency\|Coronary artery disease, association with\|Coronary heart disease\|Coronary heart disease, association with\|HDL cholesterol, association with \|Hepatitis C virus infection, association with | 1 |
| *DOCK8* | Mental retardation\|Immunodeficiency, combined\|Hyper-IgE syndrome, autosomal recessive | 1 |
| *BRCA2* | Ovarian / peritoneal carcinoma\|Oesophageal squamous cell carcinoma\|Oesophageal carcinoma \|Oesophageal cancer, association with\|Ocular melanoma\|Medulloblastoma \|Male BC risk\|Lung cancer \|Lunc cancer\|Liver cancer\|Ovarian cancer\|Ovarian carcinoma\|Ovarian insufficiency, primary \|Reactive lymphoid hyperplasia \|Prostate cancer, high-grade\|Prostate cancer \|Promyelocytic leukemia \|Potential protein deficiency\|Poorer survival in prostate cancer patients\|Peritoneal carcinoma\|Pancreatic cancer \|… | 1 |
| *LDLR* | Stroke, increased risk, association with\|Reduced plasma LDL cholesterol, association with\|Increased plasma LDL cholesterol\|Hypercholesterolaemia\|Coronary artery disease, increased risk in low BMI individuals\|Coronary artery disease, association with\|Altered transcription | 1 |
| *EDN3* | Waardenburg-Hirschsprung disease\|Waardenburg syndrome 4B\|Waardenburg syndrome 4\|Shah-Waardenburg syndrome\|Phenotype modification in HSCR\|Hirschsprung disease\|Central hypoventilation syndrome | 1 |
