# Supplementary material for: How well do whole exome sequencing results correlate with medical findings? A study of 89 Mayo Clinic Biobank samples
Source: Front Genet. 2015 Jul 24;6:244. doi: 10.3389/fgene.2015.00244 (PMC4513238; doi:10.3389/fgene.2015.00244)
Supplement: Table S7 — Number of autosomal dominant genes or dominant/recessive genes with Tier-2 single nucleotide variants for which there was a match (shown in bold) with phenotype in a Biobank participant. A total of 1091 variants of this type were noted and this was the subset with any phenotypic overlap or match. The other 1006 are not shown. [file Table7.DOCX]

**S7 Table:** Number of autosomal dominant genes or dominant/ recessive genes with Tier-2 Single Nucleotide Variants for which there was a match (shown in *bold*) with phenotype in a biobank participant. A total of 1091 variants of this type were noted and this was the subset with any phenotypic overlap or match. The other 1006 are not shown

| **Gene** | **HGMD and OMIM descriptions (some truncated)** | **Matching finding in Biobank Participant** |
| --- | --- | --- |
| ASB10 | ***Glaucoma,*** primary open angle | glaucoma |
| BFSP2 | ***Cataract***, progressive, juvenile onset\|Cataract, Y-suture\|Congenital cataract\|Diffuse cortical cataract with scattered lens opacities | cataracts |
| BRCA1 | ***Breast-ovarian cancer***, familial ; Papillary thyroid cancer, reduced risk\|Pancreatic cancer \|Pancreatic adenocarcinoma\|Ovarian carcinoma\|Ovarian cancer, association with\|Ovarian cancer\|Ovarian / peritoneal carcinoma\|Neuronal migration defect\|Mean number of breaks per cell, association with\|Peritoneal carcinoma\|… | Breast ca |
| CORIN | ***Hypertension***, association with\|Impaired brain natriuretic peptide processing | high BP |
| CRYBA4 | ***Cataract*** and microcornea\|Cataract, lamellar\|Microphthalmia | cataracts |
| DIRC2 | ***Renal cancer*** | renal ca |
| ENPP1 | Myelopathy (OPLL)\|Myelopathy (OPLL), association with\|Obesity & type 2 diabetes, association with\|***Obesity in metabolic syndrome***, association with\|Obesity, association with\|Pseudoxanthoma elasticum\|Rickets, hypophosphataemic\|Rickets, hypophosphataemic & OPLL\|Rickets, hypophosphataemic, autosomal recessive\|Major cardiovascular events in high risk individuals\|Liver damage in NAFLD\|Decreased kidney function\|Diabetes, association with \|Diabetic nephropathy, increased risk, association with\|Generalized arterial calcification of infancy\|Generalized arterial calcification of infancy and pseudoxanthoma elasticum\|Hypertriglyceridaemia in males, association with\|Hypertriglyceridemia in males, association with\|Idiopathic infantile arterial calcification\|Insulin resistance, association with | metabolic syndrome |
| EPHA2 | **Cataracts**; posterior polar, 1, 116600 (3); Cataract, age-related | cataracts |
| EPHA2 | **Cataracts**; posterior polar, 1, 116600 (3); Cataract, age-related | cataracts |
| EPHA2 | **Cataracts**; posterior polar, 1, 116600 (3); Cataract, age-related | cataracts |
| EPHA2 | **Cataracts**; posterior polar, 1, 116600 (3); Cataract, age-related | cataracts |
| ESPN | ***Hearing loss,*** non-syndromic\|Hearing loss, autosomal dominant\|Deafness and vestibular areflexia | SNHL |
| FLNC | ***Arrhythmia*** & myofibrillar myopathy, late-onset\|Distal myopathy\|Myopathy, myofibrillar | arrhythmias |
| FN1 | Glomerulopathy with fibronectin deposits\|Autism | maybe microhematuria |
| FN1 | Glomerulopathy with fibronectin deposits\|Autism | maybe microhematuria |
| KCNE2 | ***Cardiac arrhythmia***\|Long QT interval, drug induced, association with\|Long QT syndrome\|QTc interval, association with | maybe LBBB/Vtach |
| LIPI | Hypertriglyceridaemia\|Plasma HDL cholesterol\|Plasma HDL cholesterol, association with | high lipids |
| LIPI | Hypertriglyceridaemia\|Plasma HDL cholesterol\|Plasma HDL cholesterol, association with | high lipids |
| LIPI | Hypertriglyceridaemia\|Plasma HDL cholesterol\|Plasma HDL cholesterol, association with | high lipids |
| MED13L | Autism\|***Colorectal cancer***, increased risk, association with \|Congenital heart defect\|Intellectual disability, nonsyndromic, no cardiac involvement | rectal ca |
| MET | ***Papillary renal carcinoma***\|Lymphoedema \|Gastric cancer\|Diffuse large B-cell lymphoma \|Colorectal cancer \|Autism, association with | kidney cancer but not papillary type |
| MLH3 | Oesophageal cancer \|Endometrial cancer\|***Colorectal cancer***, non-polyposis\|Colorectal cancer, increased risk | colon ca |
| MSH3 | Radiosensitivity in breast cancer patients, association with\|Proximal colon cancer, increased risk, association with\|Colorectal cancer, increased risk, association with\|***Colorectal cancer*** \|Colon cancer, association with\|Colon cancer ; Endometrial carcinoma | colon ca |
| MYH6 | ***Sick sinus syndrome***, increased risk, association with\|Congenital heart defects\|Cardiomyopathy, hypertrophic\|Cardiomyopathy, dilated \|Atrial septal defect | sick sinus syndrome |
| MYPN | Cardiomyopathy, dilated\|Cardiomyopathy, dilated / ***hypertrophic\|Cardiomyopathy***, hypertrophic\|Cardiomyopathy, restrictive | possible hypertrophic cardiomyopathy |
| NEUROD1 | ***Diabetes, type 2,*** early-onset \|Diabetes, permanent neonatal\|Diabetes, MODY\|Diabetes mellitus, type 2, association with\|Diabetes mellitus, type 2\|Diabetes mellitus, type 1, association with | DM2 |
| PTCH1 | Multiple ***basal cell carcinoma*** \|Nevoid basal cell carcinoma syndrome\|Odontogenic keratocysts\|Short stature, intellectual disability & facial dysmorphism\|Skin cancer, association with\|Microcephaly and developmental delay \|Keratocystic odontogenic tumours, non-syndromic\|Basal cell carcinoma\|Gorlin syndrome \|Gorlin syndrome and autism\|Gorlin-syndrome-related odontogenic keratocysts\|Holoprosencephaly | nonmelanoma skin ca |
| RUNX1 | Thrombocytopaenia \|Thrombocytopaenia and acute myeloid leukaemia\|Thrombocytopaenia, association with\|Thrombocytopaenia, non-syndromic with ***myelodysplasia***\|Rheumatoid arthritis, susceptibility, association\|Platelet disorder, familial & myeloid leukaemia\|Platelet disorder, familial\|Mental retardation, short stature & thrombocytopaenia\|Leukaemia, chronic myelomonocytic\|Developmental delay, congenital anomalies & thrombocytopenia\|Acute myeloid leukaemia, myelodysplastic syndrome-related | yes myelodysplasia |
| AMPD1 | Adenosine monophosphate deaminase deficiency\|Features of ***metabolic syndrome*** in coronary artery disease, association with | metabolic syndrome |
| BRCA2 | Breast-ovarian cancer, familial; Fanconi anemia; **prostate** and pancreatic cancer… | prostate ca |
| BRCA2 | Breast-ovarian cancer, familial; Fanconi anemia; prostate and ***pancreatic cancer***… | pancreatic ca |
| BRCA2 | ***Breast***-ovarian cancer, familial; Fanconi anemia; prostate and pancreatic cancer… | breast ca |
| BRCA2 | ***Breast***-ovarian cancer, familial; Fanconi anemia; prostate and pancreatic cancer… | breast ca |
| CFH | Membranoproliferative glomerulonephritis\|Macular degeneration, exudative age-related, association with\|***Macular degeneration, age-related, association*** with\|Lung cancer, increased risk\|Kidney function, association with\|Inflammation, visual impairment, and cardiovascular mortality, association with\|IgA nephropathy \|Hemolysis, elevated liver enzymes & low platelet count\|Haemolytic uraemic syndrome, atypical\|Membranoproliferative glomerulonephritis, association\|Membranoproliferative glomerulonephritis, association with\|Meningococcal disease, lower risk, association with\|Thrombotic microangiopathy following transplantation\|Thrombotic microangiopathy following kidney transplantation\|Stargardt disease \|Reduced retinal sensitivity in macular region, in early age-related macular degeneration patients, association with.\|Polypoidal choroidal vasculopathy, association with\|Polypoidal choroidal vasculopathy\|No association with atypical hemolytic uraemic syndrome\|Mortality, increased risk in nonagenarians, association with\|Thrombotic thrombocytopaenic purpura, renal involvement\|Haemolytic uraemic syndrome, association with \|Haemolytic uraemic syndrome\|Coronary artery disease, early-onset, association with\|C3 glomerulonephritis.\|C3 glomerulonephritis\|Bilateral soft drusen, in age-related maculopathy, association\|AMD, tAMD, PCV and RAP, association with\|AMD, tAMD, and PCV, association with\|AMD in Asians, association with\|Alzheimer disease, association with\|Age-related macular degeneration, exudative, association with\|Dense deposit disease \|Dense deposit disease, association with\|Glomerulonephritis, crescentic and necrotizing\|Glomerulonephritis with isolated C3 deposits / Membranoproliferative glomerulonephritis\|Glomerulonephritis with isolated C3 deposits\|Glomerulonephritis \|Focal and secondary glomerulosclerosis\|Factor H deficiency\|Drusen, basal laminar\|Dense deposit disease.\|Dense deposit disease, increased risk | macular degeneration |
| FLG | ***Eczema*** \|Eczema, association with\|Eczema, association with and Asthma, association with\|Fissured skin on hands of patients without dermatitis\|Genetic modifier in pachyonychia congenita\|Hand eczema, association\|Ichthyosis vulgaris\|Peanut allergy, association with\|Psoriasis\|Psoriasis vulgaris\|Psoriasis, increased risk, association with\|Autism\|Atopic eczema \|Allergen sensitization, association with\|Atopic asthma \|Atopic asthma and dermatitis, asssociation with\|Atopic asthma, association with\|Atopic dermatitis \|Atopic dermatitis / eczema herpeticum\|Atopic dermatitis & asthma, increased risk, association with\|Atopic dermatitis and asthma\|Atopic dermatitis, increased risk, association with\|Atopic dermatitis, reduced risk, association with\|Atopic disease, association with | eczema |
| LRP6 | Fragility fractures, increased risk, association with\|Crohn's disease, early-onset ileal, association with\|Coronary artery disease, early\|Carotid artery ***atherosclerosis in hypertension, incr risk***, association\|Alzheimer disease, late onset, association with | extensive peripheral vascular disease |
| MC1R | Vitiligo protection\|UV-induced skin damage, vulnerability to\|Red hair, increased risk\|Photoaging, association with\|Melanoma, in CDKN2A mutation carriers, association with\|Melanoma, association with\|***Melanoma*** \|Increasing size of congenital melanocytic nevi, association with\|Impaired activity\|Basal cell carcinoma\|Depression, association with\|Ephelides, increased risk, association with\|Fair hair, association with\|Functional melanin, lower levels, association with\|Glucocorticoid deficiency without pigmentation | Melanoma x2 |
| NBN | Hepatic cancer, association with.\|Lung cancer, association with \|Lung cancer, increased risk, association with\|Medulloblastoma\|Melanoma\|Nasopharyngeal carcinoma, increased risk, association with\|NBS severity\|Nijmegen breakage syndrome\|Nijmegen breakage syndrome with macrocephaly, schizencephaly and large CSF spaces.\|Ovarian carcinoma\|Solid tumours\|Gastrointestinal cancer, association with\|Ganglioglioma\|Acute lymphoblastic leukaemia\|Acute lymphoblastic leukaemia, association with\|Acute lymphoblastic leukaemia, increased risk\|Aplastic anaemia\|Bladder cancer in smokers / meta-analysis, association with\|Breast cancer\|Breast cancer, increased risk, association with \|Breast cancer, reduced risk\|Cancer, increased risk, association with \|Colorectal cancer\|Fertility defects[**prostate** not listed but is in literature] | prostate ca |
| PPARG | ***Insulin resistance, diabetes*** and hypertension\|Insulin sensitivity in normoglycaemia and type 2 diabetes, association\|Maternal obesity, association with\|Obesity\|Obesity, association with\|Obstructive sleep apnea, association with\|Partial lipodystrophy\|Periodontitis, association with\|Plasma resistin levels, association with\|Polycystic ovary syndrome modifier\|Reduced serum HDL-C levels, association with\|Ulcerative colitis, association with \|Insulin resistance in type 2 diabetes, association with\|Insulin resistance\|Increased plasma leptin levels in obesity, association\|Alcoholic fatty liver disease, inflammatory, association with\|Brain tumour, increased risk \|Coronary heart disease, association with\|Crohn's disease, protection, association \|Diabetes, MODY\|Diabetes, type 2, decreased risk\|Dyslipidaemia and colorectal cancer\|Increased height/lipid metabolism, association with\|Increased body weight, association with\|Hypertension and metabolic lipid disorder, association\|Higher BMI and fat mass, association with\|Gestational diabetes and obesity, association with | DM2 |
| RP1 | ***Hypertriglyceridaemia***, association with\|Potential protein deficiency\|Retinitis pigmentosa\|Retinitis pigmentosa, autosomal recessive | high lipids |
| RP1 | ***Hypertriglyceridaemia***, association with\|Potential protein deficiency\|Retinitis pigmentosa\|Retinitis pigmentosa, autosomal recessive | high lipids |
| RP1 | ***Hypertriglyceridaemia***, association with\|Potential protein deficiency\|Retinitis pigmentosa\|Retinitis pigmentosa, autosomal recessive | hyperlipidemia |
