# Supplementary material for: How well do whole exome sequencing results correlate with medical findings? A study of 89 Mayo Clinic Biobank samples
Source: Front Genet. 2015 Jul 24;6:244. doi: 10.3389/fgene.2015.00244 (PMC4513238; doi:10.3389/fgene.2015.00244)
Supplement: Table S8 — Phenotypes of 89 individuals from chart review and the matching genotypes from WES data along with variant type, affected gene and gene inheritance information. T1S, Tier-1 SNV; T2S, Tier-2 SNV; T1I, Tier-1 INDEL; T2I, Tier-2 INDEL; CAD, coronary artery disease; DM2, diabetes mellitus type 2; CHF, congestive heart failure; CM, cardiomyopathy; SCC/BCC, squamous/basal cell carcinoma; AAA, abdominal aortic aneurysm; HCM, hypertrophic cardiomyopathy; BP, blood pressure. [file Table8.DOCX]

**S8 Table**: Phenotypes of 89 individuals from chart review and the matching genotypes from WES data along with variant type, affected gene and gene inheritance information

Acronyms - T1S: Tier-1 SNV, T2S: Tier-2 SNV, T1I: Tier-1 INDEL, T2I: Tier-2 INDEL

CAD: Coronary artery disease, DM2: Diabetes mellitus type 2, CHF: Congestive heart failure, CM: Cardiomyopathy, SCC/BCC: Squamous/Basal cell carcinoma, AAA: Abdominal aortic aneurysm, HCM: Hypertrophic cardiomyopathy, BP: Blood Pressure

| **Gender** | **Matching Trait(s)** | **Number of Diagnosis** | **# Match** | **Variant Type** | **Gene Name** | **Mode of Inheritance** |
| --- | --- | --- | --- | --- | --- | --- |
| F | DM2; Carotid Artery Stenosis & CAD; CAD; Osteopenia | 13 | 4 | T2S; T2I | *ACACB ADD1 MYH15 ASPN* | SNP |
| M | prostate cancer; high BP | 17 | 2 | T2S | *HIF1A; STK39* | SNP; AD/AR; SNP |
| F | Atherosclerosis; HCM; Macular degeneration; Osteoporosis; high lipids | 15 | 5 | T1S; T1S; T2S | *MSR1 AGTR2 CFH ESR1 MYPN RP1 TTN* | ADMono; XL-R & ADMono; AD/AR; SNP; AD/AR |
| M | high BP; CHF; DM2; Left ventricular dysfunction and DM2 | 10 | 4 | T1S; T2Sx3 | *ACSM3 CLCNKA MTMR9 PPARGC1A* | SNP; digenic; SNPx2 |
| M | Depression; high BP | 7 | 2 | T1S; T2S | *TPH1 GRK4 KDR* | SNP |
| F | Arrhythmias | 15 | 1 | T2S | *FLNC* | ADMono |
| F | Rheumatoid arthritis | 10 | 1 | T1S; T2S | *CCL26; PTPN22* | SNP |
| M | No Match | 9 | 0 | NA | NA | NA |
| F | No Match | 8 | 0 | NA | NA | NA |
| F | high lipids | 12 | 1 | T2S | *CYP1A1* | SNP |
| F | DM2 | 8 | 1 | T2S | *THADA* | SNP |
| M | Metabolic syndrome | 5 | 1 | T2S | *STEAP4* | SNP |
| M | high lipids | 18 | 1 | T2S | *ACAT2* | SNP |
| M | Obesity; Prostate cancer | 5 | 2 | T2S | *ACACB NBN* | SNP; AD/AR |
| M | Diabetes & Obesity; Hyperlipidemia; colon cancer; DM2 | 6 | 4 | T2S | *ACACB APOA4 BIRC5 ENPP1 EXO1 MFI2 MLH3 MSH3 MYO18B* | SNP; SNP; ADMono & SNP; ADMono |
| M | Cardiometabolic risk traits; Nephrosclerosis and diabetes; MI; cardiomyopathy but likely hypertensive CM; high BP | 8 | 5 | T2S; T1I; T2I | *CHGA GHRL MIA3 PSMB9 SCG2 SYNM STK39* | SNP |
| F | high lipids; Cataracts; CAD; Chronic renal failure | 9 | 4 | T2S; T1I | *APOA4 BFSP2 CREB3L3 EPHA2 MYH15 CFHR5* | SNP; ADMono; SNP; ADMono |
| F | CAD; Glaucoma | 11 | 2 | T2S | *LPA MYH15 WDR36* | SNP |
| M | Prostate cancer | 9 | 1 | T2S | *CTSB* | SNP |
| M | Eczema; Obesity; SCC/BCC; Metabolic syndrome | 9 | 4 | T1S; T2S | *FLG; ACACB; CYP4A11; CYSL;TR2; IGFBP5; SHBG* | AD/AR; SNP |
| M | Lung cancer; Cataracts | 11 | 2 | T2S | *COL12A1; EPHA2* | SNP; ADMono |
| F | Glaucoma; Cataracts | 7 | 2 | T1S; T2S | *NT5C1B; DMXL1; EPHA2* | SNP; ADMono |
| M | DM2; Prostate cancer; Extensive peripheral vascular disease; Lung cancer | 8 | 4 | T2S | *ADIPOQ BRCA2 CD82 LRP6 MAD1L1 TP53AIP1* | SNP; AD/AD & SNP; AD/AR; SNP |
| F | DM2 | 10 | 1 | T2S | *PPP1R3A* | digenic |
| M | Lung cancer | 6 | 1 | T2S | *ITGA11; MS4A6A* | SNP |
| M | DM2 | 13 | 1 | T2S | *ACACB; NEUROD1* | SNP & ADMono |
| F | Osteopenia | 14 | 1 | T1S | *ESR2* | SNP |
| M | high lipids; high BP; Chronic renal failure | 10 | 3 | T2S; T1I; T2I | *LIPE SLC14A2 CFHR5 APOB* | SNP & AD/AR; SNP; ADMono |
| M | No Match | 6 | 0 | NA | NA | NA |
| M | Glaucoma; Myelodysplasia | 11 | 2 | T1S; T2S | *TULP3; RUNX1* | ADMono_CNV; ADMono |
| F | Depression; high lipids; Thyroid | 9 | 3 | T2S; T2I | *ABCA13 NPC1L1 FOXE1* | SNPx2; AR |
| M | No Match | 15 | 0 | NA | NA | NA |
| M | high BP, high glucose | 5 | 2 | T2S | *ADAMTS16; MC1R; PON1* | SNP; AD/AR; SNP |
| M | Hearing loss; Non-melanoma skin cancer | 6 | 2 | T2S | *PDZD7 PTCH1 TMPRSS5 TSPEAR* | SNP & AR; ADMono |
| M | No Match | 12 | 0 | NA | NA | NA |
| M | high lipids | 10 | 1 | T1S | *LIPC* | AR |
| F | Osteopenia; high lipids | 8 | 2 | T2S; T2I | *C17orf53 RP1 APOB* | SNP; AD/AR |
| M | Depression; Lung cancer | 16 | 2 | T1S; T2S | *ABCA13; ABCB1; HUS1B; TP53BP1* | SNP |
| M | No Match | 11 | 0 | NA | NA | NA |
| M | Vitreous detachment; Cataracts | 11 | 2 | T1S; T2S | *LEPREL1; CRYBA4* | AR; ADMono |
| M | AAA and pulmonary fibrosis | 8 | 2 | T1S; T2S | *SMAD3; MUC5B* | ADMono; SNP |
| F | Rheumatoid arthritis | 11 | 1 | T2S; T1I | *MICA* | SNP |
| M | No Match | 7 | 0 | NA | NA | NA |
| F | Chronic Obstructive Pulmonary Disease; Breast cancer; DM2; Hearing loss | 12 | 4 | T2S; T1I; T2I | *A2M PML PPARG RAD54L GJB2 GSPT1* | SNP; SNP; AD/AR; ADMono |
| F | Cancer | 5 | 1 | T2S | *GSPT1* | SNP |
| F | No Match | 8 | 0 | NA | NA | NA |
| M | Depression; Glaucoma; Prostate cancer; Hearing loss | 17 | 4 | T2S; T1I | *ABCA13 ACACB ASB10 CTSB OPTC SLIT3 GJB4* | SNP; ADMono; SNP; AD/AR |
| M | DM2; Lung cancer; high lipids; CAD | 8 | 4 | T2S; T1I | *ATF6 IGFBP5 LIPE LIPI SNX19 XRCC3* | SNPx3; ADMono; SNP |
| M | Depression; high BP; DM2 | 6 | 3 | T2S | *ABCA13 ACSM3 THADA* | SNP |
| M | No Match | 12 | 0 | NA | NA | NA |
| F | Pancreatic cancer | 7 | 1 | T2S | *ADH1B BRCA2* | SNP & AD/AR |
| M | Multiple sclerosis | 7 | 1 | T1S | *HLA-DRB1* | SNP |
| F | Breast cancer | 2 | 1 | T2S | *BRCA1* | ADMono |
| M | high BP | 5 | 1 | T2S | *ACSM3* | SNP |
| M | No Match | 4 | 0 | NA | NA | NA |
| M | Metabolic syndrome; DM2 | 14 | 1 | T2S; T2I | *AMPD1; THADA* | AD/AR; SNP |
| F | Hyperlipidemia; high BP | 6 | 2 | T2S; T2I | *SREBF2 KCNMA1* | SNP |
| F | Hyperlipidemia | 5 | 1 | T2S | *ITIH4* | SNP |
| M | No Match | 8 | 0 | NA | NA | NA |
| M | DM2 | 6 | 1 | T1S | *PIK3C2G* | SNP |
| M | Renal cancer; Sensorineural hearing loss; Eczema; Kidney cancer but not papillary type; Hyperlipidemia; prostate cancer | 8 | 7 | T2S | *DIRC2 ESPN FLG GSPT1 MET RP1 TLR10* | ADMonox2; AD/AR; SNP; ADMono; AD/AR; SNP |
| M | high BP | 11 | 1 | T2S | *NPR1* | SNP |
| F | Breast cancer | 10 | 1 | T2S; T2I | *AKAP13; GSPT1* | SNP |
| F | No Match | 7 | 0 | NA | NA | NA |
| F | high BP, high lipids | 8 | 2 | T2S | *GRK4; LIPI* | SNP; ADMono |
| M | DM2 | 8 | 1 | T2S | *CD38* | SNP |
| M | Hearing loss; CAD; high BP | 12 | 3 | T2S; T2I | *CDH23 MIA3 SLC14A2 ESPN* | digenic & ADMono; SNPx2 |
| M | DM2; Metabolic syndrome; Hearing loss; high lipids | 9 | 4 | T1S; T2S; T1I; T2I | *PIK3C2G FN3K MTMR9 MYO1C SLC30A8 GPR98 APOB* | SNPx3; digenic; AD/AR |
| F | Cataracts | 6 | 1 | T2S | *EPHA2* | ADMono |
| M | No Match | 6 | 0 | NA | NA | NA |
| F | No Match | 2 | 0 | NA | NA | NA |
| M | Graves disease; Immunocomplex glomerulonephritis | 6 | 2 | T1S; T2S | *HLA-DRB1; C3AR1* | SNP |
| M | Psoriasis | 10 | 1 | T2S | *ZNF750* | SNV |
| M | No Match | 0 | 0 | NA | NA | NA |
| F | Colon cancer | 7 | 1 | T1I; T2I | *NQO1 GSPT1 EPHB6* | SNP |
| M | Sick sinus syndrome | 8 | 1 | T2S | *MYH6* | ADMono |
| M | CAD | 6 | 1 | T2S | *ADIPOQ* | SNP |
| F | high BP, high lipids | 7 | 2 | T2S | *CORIN; EPHX2* | ADMono; SNP |
| M | high BP | 9 | 1 | T2S | *SLC14A2* | SNP |
| F | Mixed connective tissue disease; Lung disease | 3 | 2 | T2S; T2I | *ITPR3 MYLK* | SNP; ADMono |
| F | DM2 | 6 | 1 | T2S | *TGM2* | SNP |
| M | Psoriasis; Rectal cancer | 2 | 2 | T2S; T2I | *HLA-C HLA-C MED13L MYO18B GSPT1* | SNPx2; ADMono & SNPx2 |
| F | Rheumatoid arthritis; Cataracts | 11 | 2 | T1S; T2I | *CCL26 CTDP1* | SNP; AR |
| F | Bladder cancer | 8 | 1 | T2I | *SLC23A1* | SNP |
| F | Ventricular tachycardia but with known CAD | 9 | 1 | T1I | *DSC2* | ADMono |
| F | Retinal vein occlusion | 4 | 1 | T2S | *SERPINC1* | AR |
| F | Breast cancer | 1 | 1 | T2S | *BRCA2* | AD/AR |
| F | No Match | 8 | 0 | NA | NA | NA |
| F | low vitamin B12; high BP | 6 | 2 | T2S; T2I | *TCN1 STK39* | AR; SNP |
