# Supplementary material for: How well do whole exome sequencing results correlate with medical findings? A study of 89 Mayo Clinic Biobank samples
Source: Front Genet. 2015 Jul 24;6:244. doi: 10.3389/fgene.2015.00244 (PMC4513238; doi:10.3389/fgene.2015.00244)
Supplement: Table S9 — Distribution of 89 WES samples by cancer diagnosis and gender. Also included are metrics on Tier-1/Tier-2 SNV and INDEL along with the list of cancer predisposition genes found in the groups. [file Table9.DOCX]

**S9 Table**: Distribution of 89 WES samples by cancer diagnosis and gender. Also included are metrics on Tier-1 / Tier-2 SNV & INDEL along with the list of cancer predisposition genes found in the groups.

|  | **89 Biobank Samples** | | | |
| --- | --- | --- | --- | --- |
|  | **Cancer diagnosed** | | **No cancer diagnosed** | |
| **# of samples** | 39 | | 50 | |
| **Total Variants** | 58 | | 57 | |
| **Mean, Range** | 1.3, 0-3 | | 1.1, 0-4 | |
| **SNV** | 54 | | 57 | |
| **INDEL** | 4 | | 0 | |
| **Cancer Genes** | 24 | | 20 | |
|  | **Male** | **Female** | **Male** | **Female** |
| **# of samples** | 26 | 13 | 25 | 25 |
| **Total Variants** | 41 | 17 | 31 | 26 |
| **Mean, Range** | 1.6, 0-3 | 1.3, 0-3 | 1.2, 0-4 | 1, 0-3 |
| **SNV** | 39 | 15 | 31 | 26 |
| **INDEL** | 2 | 2 | 0 | 0 |
| **Cancer Genes** | 20 | 10 | 19 | 13 |
| **Lists of Cancer Genes** | APC | ATM | APC | APC |
|  | ATM | ATR | ATM | ATM |
|  | BRCA1 | BARD1 | ATR | ATR |
|  | BRCA2 | BMPR1A | BRCA1 | BARD1 |
|  | BRIP1 | BRCA1 | BRCA2 | BRCA1 |
|  | CDKN2A | BRCA2 | CDH1 | BRCA2 |
|  | FAM175A | MUTYH | CDKN2A | GALNT12 |
|  | FLCN | PALB2 | CHEK2 | MRE11A |
|  | GALNT12 | TP53BP1 | GALNT12 | NBN |
|  | MSH6 | TSC1 | MRE11A | PALB2 |
|  | MUTYH |  | MUTYH | PRSS1 |
|  | NBN |  | NBN | RAD50 |
|  | PALB2 |  | PALB2 | TSC2 |
|  | POLE |  | POLE |  |
|  | PRSS1 |  | PRSS1 |  |
|  | RAD50 |  | RAD50 |  |
|  | RAD51C |  | RAD51C |  |
|  | SMAD4 |  | TP53BP1 |  |
|  | TP53BP1 |  | TSC2 |  |
|  | TSC2 |  |  |  |
